# COVID-19 INFORMATION ELICITING STEREOTYPE THREAT CAN HARM OLDER ADULTS’ COGNITIVE FUNCTION

**DOI:** 10.1093/geroni/igad104.3253

**Published:** 2023-12-21

**Authors:** Julie Pham, Morgan Shumaker, Holly Bowen

**Affiliations:** Southern Methodist University, Dallas, Texas, United States; Texas Christian University, Fort Worth, Texas, United States; Southern Methodist University, Dallas, Texas, United States

## Abstract

During the COVID-19 pandemic, older adults were identified as an at-risk group for developing severe physical symptoms. In addition to physical presentations, evidence indicated COVID-19 related changes in cognitive and neurological function; specifically, increased risk of stroke, reduced attention, concentration, and memory problems. Given that older adults remain particularly vulnerable to severe symptoms, exposure to information about COVID-19 and the associated cognitive symptoms may have adverse effects on their cognitive performance. This phenomenon, known as stereotype threat, describes situations in which negative stereotypes about a particular group can impair functioning. The current study aims to examine how exposure to adverse age-related information about the pandemic may impact cognitive performance compared to more neutral age-related information. 484 older adults were recruited February through July 2023 via Amazon Mechanical Turk and Cloud Research Connect. Participants were randomly assigned to a neutral or threat condition and read a paragraph related to aging or COVID-19, respectively. Afterwards, participants completed a memory task followed by a series of questionnaires to assess demographics, mood, depression, and COVID-19 health history. A one-way ANCOVA was conducted to evaluate the effect of COVID-19 threat on memory performance while controlling for history of COVID diagnosis. Findings indicate a significant difference in scores such that individuals in the threat condition had worse memory performance, compared to those in the neutral condition. This suggests that just reading information about COVID-19 may pose a threat to older adults’ cognitive abilities and further demonstrates the far-reaching effect the pandemic has had on individuals.

